# Extreme Female Promiscuity in a Non-Social Invertebrate Species

**DOI:** 10.1371/journal.pone.0009640

**Published:** 2010-03-11

**Authors:** Marina Panova, Johan Boström, Tobias Hofving, Therese Areskoug, Anders Eriksson, Bernhard Mehlig, Tuuli Mäkinen, Carl André, Kerstin Johannesson

**Affiliations:** 1 Department of Marine Ecology-Tjärnö, University of Gothenburg, Strömstad, Sweden; 2 Department of Physics, University of Gothenburg, Göteborg, Sweden; 3 University of Alaska Museum of the North, Fairbanks, Alaska, United States of America; McGill University, Canada

## Abstract

**Background:**

While males usually benefit from as many matings as possible, females often evolve various methods of resistance to matings. The prevalent explanation for this is that the cost of additional matings exceeds the benefits of receiving sperm from a large number of males. Here we demonstrate, however, a strongly deviating pattern of polyandry.

**Methodology/Principal Findings:**

We analysed paternity in the marine snail *Littorina saxatilis* by genotyping large clutches (53–79) of offspring from four females sampled in their natural habitats. We found evidence of extreme promiscuity with 15–23 males having sired the offspring of each female within the same mating period.

**Conclusions/Significance:**

Such a high level of promiscuity has previously only been observed in a few species of social insects. We argue that genetic bet-hedging (as has been suggested earlier) is unlikely to explain such extreme polyandry. Instead we propose that these high levels are examples of convenience polyandry: females accept high numbers of matings if costs of refusing males are higher than costs of accepting superfluous matings.

## Introduction

In many species, females mate with more than one male to decrease their risk of only receiving sperm of poor quality or with low compatibility, to increase the probability of receiving sperm with sexually selected “good genes”, to avoid inbreeding and to increase genetic diversity of the offspring [1]–[6]. However, benefits of multiple matings are likely to decline with number of males owing to sampling effects, that is, additional matings will only marginally contribute with genes of better quality [2], unless postcopulatory mate-choice, such as cryptic female choice and sperm competition, is extremely effective [5]. Moreover, benefits of matings are traded off against costs of additional matings [1]. These are possible reasons why, in a majority of studied species, multiple mating means that a female mates with more than one but seldom more than a few males [3], [7]–[10]. Nevertheless, in some species of single-queen social insects and high-density flies, females mate ten times or more as many males than in other species [11]–[13], which may be explained by increased genetic variation among offspring [14], nutritional benefits [15]–[16], [ but see 17]–[18 for examples of where nuptial gifts instead have detrimental effects on females], or convenience polyandry where costs of resisting matings exceed the costs of additional matings [19].

Female *L. saxatilis* become mature after six months and can live several years. Mating activity is most intense during spring and summer but copulating pairs can be observed year round. The species is ovoviviparous and the female retains the fertilized eggs in a brood-pouch until they hatch into 0.5 mm small snails. Populations are dense (100–1000/m^2^) and sex ratios are even. Consequently, most males encounter tens of receptive females each day. Experimental studies show no evidence of precopulatory female-based mate-choice and no male-male competition, although males can exert choice with respect to size of partners [20]. To find a partner, the male follow the mucous trail of other snails [21]. When encountering a female, he mounts her shell and positions himself at the right hand side, inserting the penis under the shell of the female. Males mount shells of both females and males, as well as juveniles, but copulation attempts with males and juveniles are interrupted after only a few minutes, while matings with females lasts 20–30 minutes or more [20], [22]. During copulation the female is usually inactive, making no attempts to reject the male [22]. Sperm is slowly transferred by ciliary movement along the groove in the male penis into *bursa copulatrix* of the female [20]. Besides fertile eusperm, ejaculates contain parasperm - sterile germ cells that have lost their nucleus, and are thought to facilitate transport of the eusperm [23]. During mating the female carries the male, which increases the risk of her being dislodged by waves and translocated from the upper littoral zone to the sublittoral where predation by crabs and fishes are much more severe (Johannesson et al. subm.). Females carry mature offspring in their brood pouch throughout the year. When females are kept in isolation, they continue to give birth to new offspring for at least ten months (Johannesson pers. obs.), which indicates that they have the capacity to store functional sperm for long times.

The reproductive biology of *Littorina saxatilis* suggests that females may be promiscuous and that multiple paternity is common. In an earlier attempt to estimate the level of multiple paternity in *L. saxatilis* we detected an average of 7.6 males contributing to broods of wild-mated females [24]. However, we analysed only 20–23 out of 23 to 87 offspring per brood. Based on earlier results of about five sires per clutch in a related species *Littorina obtusata* [25], we expected this to be enough to estimate the number of sires. Since we estimated up to ten sires in one sample, the question arose whether the sample size used allowed us to detect all sires in a brood. The true or most likely number of sires in a brood could not be estimated directly or by modeling from these results, since, theoretically, sampling and analyzing more offspring may produce either a distribution with a higher number of offspring per sire but the same number of sires in the brood, or the same average number of offspring per sire and more sires in each brood.

To estimate the true paternity level in *L. saxatilis* we now genotyped almost all offspring in four large broods from our previous experiment. The results show that the number of sires involved increased substantially, whereas the average number of offspring per sire was similar to what was found using a smaller sample size. This new finding challenges our earlier suggestion that polyandry in *L. saxatilis* is a result of genetic bet-hedging applied by the females to avoid inbreeding, bad genes and genetic incompatibility [24].

## Methods

### Sampling the Broods

The experiment was performed in 2004 and part of the material was earlier presented in [24] where a detailed description of the experimental design can be found. In brief, 18 wild-mated females were collected in July on the island Saltö (N58°53′, E11°10°) and incubated during two and a half months in small aquaria with a constant flow-through of seawater. At the end of this period 15 females were found to have produced 23–302 offspring (one female died and two did not produce any offspring). All females and offspring were subsequently stored at −70°C. In the previous study we randomly chose eight families with total clutch sizes of 23–87, and analysed 20–23 randomly chosen offspring per family. In the present study we analysed more offspring from three of the earlier analysed families: F5, F7 and F8, that had intermediate clutch sizes (69–87), and also included a new family F9, containing 117 offspring. After combining the previous and new data, the number of analysed offspring was 53–79 per clutch (Table 1). DNA extraction was not successful for the smallest juveniles.

**Table 1:** The number of sires in four half-sib families of *Littorina saxatilis*.

Female	Observed no of offspring	Analysed no of offspring	Most likely no of sires	Minimum no of sires
F2	87	77	23	21
F6	71	71	16	15
F8	69	53	15	12
F9	117	79	23	20

Four females and their offspring were genotyped at five microsatellite DNA loci. The most likely number was estimated using the likelihood-based software COLONY and the minimum number was calculated using MINSIRES.

### Microsatellite Genotyping

DNA was extracted by the CTAB method [26], using pieces of muscle tissue from the females and whole juveniles. Five microsatellite loci, *Lsub62*, *Lsub32*, *Lsub8* [27], *Lsax6* and *Lx23* [28] were amplified following the PCR protocol described in [24]. Allele sizes were determined by electrophoresis on a Beckman Coulter CEQ 8000 automatic sequencer, followed by analysis with the CEQ Fragment Analysis software.

### Paternity Inference

The likelihood-based software COLONY [29] was used to divide clutches into full-sib families and to estimate genotypes of sires. Population allele frequencies were calculated from the data, including the families analysed earlier [24], for more accurate estimation. In the analyses, we assumed that only females were polygamous, since COLONY allows either polygyny or polyandry, not both. However, given the high population density and low motility of the snails and that the females were sampled several meters apart, it is very unlikely that the same male would have mated with two or more of the analysed females. Genotyping error rate was set at 2%, as estimated from the frequency of cases when the genotype of an offspring and the mother did not match (in such cases the genotype in that locus was denoted as missing data). An earlier analysis of inheritance of the microsatellite loci used in this study showed the presence of null alleles in the loci *Lsax6* and *Lsub8* but no evidence of allele drop out in any of the loci [30]. However, the observed rare mismatches could not be explained by maternal null alleles, since maternal genotypes were heterozygous, with both alleles segregating in the rest of the offspring in a Mendelian fashion. Instead, several different sources (stuttering, amplification of non-specific fragments) appeared to contribute to the genotyping artefacts. Accordingly, we ran COLONY with “other than drop out” error set at 2% rate per locus. This analysis was run three times, and the configuration with best Log Likelihood was chosen for further analyses. The most likely paternal configurations were also compared with the minimum numbers of sires explaining offspring genotypes in each half-sib family, obtained using MINSIRES, a software that calculates the minimum number of sires from multi-locus genotypes of progeny in cases with large number of sires per brood [31].

The observed numbers of offspring per sire, as estimated by COLONY, were compared with the distribution expected under random mating in a large gamete pool, using a truncated Poisson distribution [29], [32]. To test whether genetic similarity between parents affects fertilization success, we estimated the correlation between the number of offspring and the relatedness between parents [33] with the software RELATEDNESS 5.0.8 (http://www.gsoftnet.us/GSoft.html) using maternal and estimated paternal genotypes. In both the analysis of random mating and genetic similarity, the data for the four families were pooled.

To assess the effect of sample size on the estimated number of sires, we subsampled offspring of one randomly chosen female (F2) and estimated the most likely number of fathers. This was repeated 55 times with sample sizes ranging between 10 and 77 juveniles.

## Results

The five microsatellite loci displayed high levels of polymorphism, with numbers of detected alleles per locus ranging between 9–23. The most likely numbers of sires contributing to each of the families were 23, 16, 15 and 23, as estimated by COLONY (Table 1). The results of the three independent runs showed very little variation: number of sires was estimated to 14 instead of 15 in F8 in one run and to 24 instead of 23 in F9 in another. Furthermore, high proportions of the offspring were assigned to identical full-sib families in all three runs (68, 100, 94 and 92% for the F2, F6, F8 and F9 families, respectively). Estimating the true least number of fathers based on multi-locus genotypes of the progeny in MINSIRES [31] gave minimum numbers of sires of 21, 15, 12 and 20, which are all very close to the estimated full number of sires that we obtained from the likelihood approach using COLONY, thus confirming the high level of multiple paternity. Indeed, the two estimates are expected to be close when the true number of sires is large [31].

**Figure 1:**
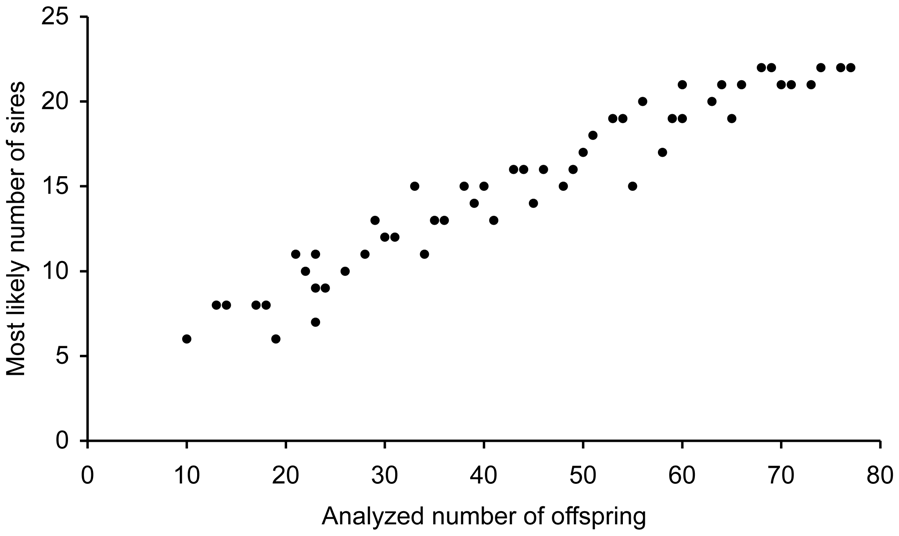
The effect of sample size on the estimated number of sires. Most likely number of sires is estimated using COLONY in random subsamples of offspring from a single brood of *Littorina saxatilis*; subsample size varies from 10 to 77 (i.e. the whole brood).

**Figure 2:**
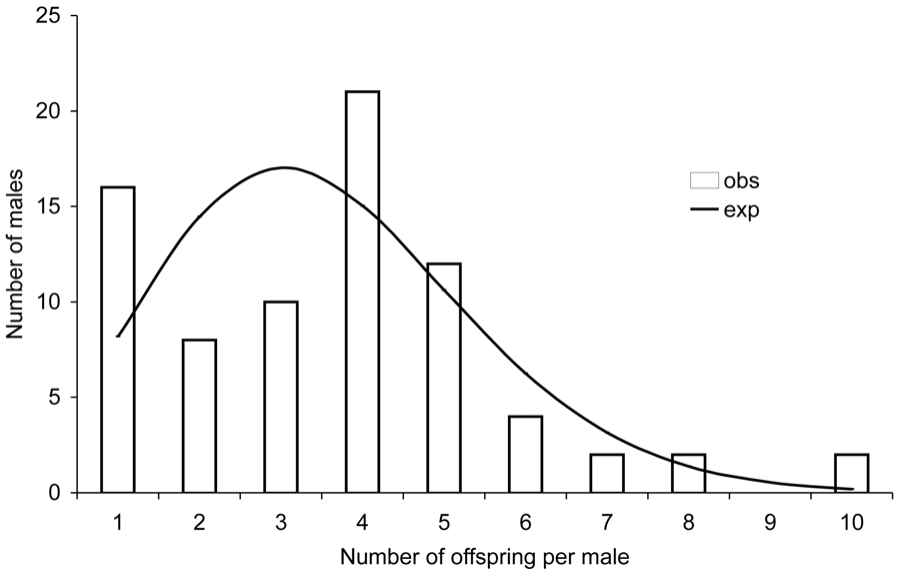
Full-sib family size distribution in four broods of *Littorina saxatilis*. Observed number of offspring per male is estimated in COLONY. Expected number of sires is approximated by a truncated Poisson distribution.

The repeated subsampling of various numbers offspring demonstrates that the higher level of multiple paternity in the present study (mean ± S.E. = 19.3±2.2), as compared with the earlier results (mean ± S.E. = 7.6±2.1) [24] is a matter of sample size (Fig. 1). Within each half-sib family each father contributed to between one and eleven offspring, with a mean number of offspring per father of 3.6 (Fig. 2), which is close to 2.9, found in the previous study. A truncated Poisson distribution overlaying the distribution of paternal offspring suggests that the fertilization process deviates from a random process (*P* = 0.005, *df* = 5, χ^2^ = 16.7), with more males than expected by chance siring one or four offspring and less males than expected siring two or three offspring (Fig. 2). In the three families that were partially analysed earlier, most of the full-sib families increased in size by adding more data while only a few of the new offspring resulted in new full-sib families (with only one member). This argues against the possibility that the number of sires increased merely due to higher possible number of genotyping errors in the larger dataset. Finally, we found no correlation between relatedness of male to the female and the number of offspring sired by this male (*R* ^2^ = 0.017; *P* = 0.26). However, this result should be treated with caution, since many sires had 1–4 offspring only, and thus, their genotypes could not be reconstructed with high confidence (average probabilities ± S.E. for locus genotypes of sires with 1, 2, 3, 4 and 5 or more offspring were 0.26±0.02, 0.53±0.04; 0.72±0.04, 0.81±0.02 and 0.92±0.02, correspondingly).

## Discussion

We have shown that polyandry in the marine snail *L. saxatilis* is exceptionally high, in fact among the highest ever recorded. In *L. saxatilis*, a brood of 60–80 offspring from one female is sired by 15–23 males, each male being the father of only 5–6% of the offspring. We acknowledge two potential limitations in the present dataset: small sample size (four clutches) and the presence of null alleles in two of the five microsatellite loci used for genotyping. However, it is not likely that these factors inflated our estimates of number of sires in the snail families. Variation in the number of sires between the four clutches was relatively low compared to the mean (mean ± S.E = 19.3±2.2), and in the earlier analysis of eight clutches the number of sires did not vary much either [24]. Thus, it seems unlikely that, by chance, we picked four extremely promiscuous females; however, for more precise estimates of multiple paternity levels and its variation between clutches in this species a larger sample size is warranted. Null alleles might cause a problem in paternity reconstruction from genetic data, producing apparent mismatches between genotypes of parents and their offspring. While we did not detect any maternal null alleles in the analyzed families, there could still be sires with null alleles in two of the analyzed loci. This was taken into account by allowing for genotyping errors in paternity reconstruction in COLONY, i.e. mismatch in a single locus did not lead to an immediate inference of an additional sire [29].

Although promiscuity is common among animal species, such extreme levels of multiple paternity as reported here for *L. saxatilis* have rarely been found outside a few species of social insects in which new colonies are established by single queens [13], [34], [35]. In addition, extreme levels of multiple mating have been reported for some other insect species, such as the seaweed fly (*Coelopa frigida*) in which a female may mate 600 males during her three week life [36]; genotype data is needed, however, to test if this corresponds to similarly extreme levels of multiple paternity.

In a range of species, female promiscuity is used as an effective tool to avoid inbreeding [37]–[40], or to reduce risks of genetic incompatibility [41], [42]. In most promiscuous species studied, however, only two or a few males are involved in siring offspring of the same female during the same mating season [3]. These findings fits the general prediction from theory, that female costs increase linearly with the number of mated males, while female rewards peak at a low number of mated males and thereafter decline asymptotically [2], [43]. Consequently, extreme levels of polyandry are unlikely to add genetic benefits to the female and her offspring, unless circumstances are exceptional such as in some species of social insects where effective population sizes are severely restricted by a large part of each colony being excluded from reproduction, in particular in species that found new colonies by single mated queens. Indeed, modelling shows that genetic bet-hedging is not a successful strategy unless populations are small and costs of mating are low [14]. This model is supported by recent empirical data: polyandry involving 2–4 males did not increase levels of genetic variation and/or fitness of offspring in a shark species in comparison to monoandry [10]; similarly, genetic variation increased only marginally due to polyandry (2–3 males) in a species of social ants [13]. Hence, recent findings challenge earlier opinions that genetic benefits are important to explain multiple paternity (although see [44]). Consequently, extreme levels of polyandry are even more difficult to explain with genetic benefits for the female and the offspring. The finding that the number of sires of a brood of *L. saxatilis* is as high as 15–23 thus challenges our earlier suggestion that genetic bet-hedging is a main explanation for polyandry in this species [24].

An alternative explanation for promiscuity is convenience polyandry [19], that is, females take the costs of additional matings instead of spending time and energy on trying to reject harassing males. Convenience polyandry is documented among species of reptiles, insects and crustaceans, often in combination with male-female battles, and sometimes with cryptic female choice [36], [45]–[47]. In seaweed flies, high population densities together with an even sex ratio and intense precopulatory female-male battles, suggests that female flies take the costs of superfluous matings instead of increased costs of prolonged battles [48]. Similarly, *L. saxatilis* forms exceptionally dense populations of both sexes on the shore, increasing a female's risk of male harassment. In contrast to the flies, females rarely attempt to reject mounting males [22], despite the fact that matings are costly to females. In a recent study we show that females that carry males during mating are more susceptible to dislodgement by waves than females without males on their back, (Johannesson et al. subm). A likely explanation is that costs for rejection of males are higher than accepting superfluous matings. To withdraw into the shell, for example, will result in dislodgement and likely being washed off shore with a substantially increased mortality risk [49]. Instead of rejecting mounting males, females actively halve the number of matings by producing an andromorphous mucous trail that result in males being unable to discriminate between female and male trails of this species, as is possible in closely related species (Johannesson et al. subm.). This is a very cost-effective way of reducing the number of matings.

The fact that the distribution of number of offspring per sire deviated from the expected number under random fertilization would suggest postcopulatory sexual selection in the species. However, as sires with one or four offspring were over-represented and sires with two or three offspring were under-represented, the results are inconclusive and a further experimental assessment is needed to test for postcopulatory sexual selection. Although we did not find a correlation between relatedness of male and female genotypes and number of offspring, the possibility that some sperm were selected against remains open due to obvious limitations of inferences from offspring of wild-mated females: 1) there could have been matings with additional males that did not result in any offspring; 2) genotypes of sires that had 1–2 offspring cannot be reconstructed reliably. This will be investigated further in a laboratory experiment when virgin females are mated with several known males.

Another possibility is if snail females benefit from multiple matings by receiving nuptial gift in the form of parasperm [23], [24]. The general function of parasperm is paternity assurance that in different species of insects includes nuptial gifts to females, suppressing female mating activity, formation of sperm plugs after copulation or even attacks on the rival sperm [15]–[18]. Although prosobranch molluscs exhibit complex and diverse forms of parasperm, their function is still unknown [23]. In *Littorina* snails, parasperm cells produce polysaccharide-rich vesicles, released in the female *bursa copulatrix*, which could serve as nuptial gifts, and lysosomes with other secretory products that may affect the female or the rival sperm [23], [50]. However, these hypotheses are solely based on cell morphology of the parasperm and require experimental support [23], [50].

As discussed earlier [24], *L. saxatilis* may pass severe population bottlenecks associated to occasional toxic algal blooms and recolonization of island sites. During such events polyandry would potentially be beneficial to females colonizing new sites, similar to queens of social insects that establish a new colony. Indeed, high levels of multiple paternity (ten sires per brood or more) substantially increase effective population size compared to low levels of multiple paternity, for which the effective population size is actually lower than for complete random mating and monogamy [51], [52]. Possibly, the extreme level of polyandry in *L. saxatilis* is beneficial during occasional bottlenecks, but the costs of multiple mating will still be carried by all females daily, so this is unlikely to be the sole explanation for this unique behaviour. Instead, we conclude that convenience polyandry is a main factor explaining the extreme level of polyandry in *L. saxatilis*, but that positive genetic effects during situations of strong bottlenecks, nutritional benefits and postcopulatory sexual selection may add to pay for the increased costs of excessive matings.
